# Customizing Your Demons: Anxiety Reduction via Anthropomorphizing and Destroying an “Anxiety Avatar”

**DOI:** 10.3389/fpsyg.2020.566682

**Published:** 2020-12-03

**Authors:** Daniel Pimentel, Sri Kalyanaraman

**Affiliations:** ^1^Oregon Reality Lab, School of Journalism and Communication, University of Oregon, Portland, OR, United States; ^2^Media Effects and Technology Lab, College of Journalism and Communications, University of Florida, Gainesville, FL, United States

**Keywords:** customization, avatar, anxiety, self-discrepancy theory, anthropomorphism, mental health

## Abstract

Character customization is a prominent feature in digital games, affording users the ability to tailor one’s virtual self-representation (avatar) to match aspects of their actual or ideal self, influencing psychological well-being. The mental health implications of character customization can be partially explained by self-discrepancy theory, which argues that achieving congruence with one’s avatar reduces cognitive dissonance. However, the role of undesirable self-concepts such as mental health ailments have largely been overlooked in this context despite forming part of one’s identity. In theory, customization of an avatar representing undesirable self-concepts presents a self-regulatory paradox: individuals desire to reduce discrepancies with a self-representation, yet they also desire to enlarge discrepancies with a disliked-self. To reconcile this, two experiments explored the psychological implications of imbuing avatars with undesirable self-concepts. In Study 1 (*N* = 90), participants customized an avatar to represent anxiety within themselves (i.e., an anxiety avatar). Customization significantly reduced state anxiety compared to a control group, supporting the proposed discrepancy-reduction mechanism. Study 2 (*N* = 122) employed a 2 (customization: yes, no) × 2 (destruction: yes, no) between-subjects design, with participants either destroying or observing an anxiety avatar. Destruction of customized anxiety avatars resulted in the largest reduction in anxiety among all conditions, supporting the proposed discrepancy-enlargement mechanism. Theoretical and practical implications for the use of avatar-based e-mental health interventions are discussed.

## Introduction

In 2016, United Kingdom-based illustrator Toby Allen’s “Real Monsters” series depicted mental health ailments (e.g., anxiety) as anthropomorphic, or humanlike, creatures. The artwork received widespread publicity on various online communities for raising awareness of mental health issues. However, despite being lauded by these communities, some commenters expressed desires to visually modify the creatures to better align with their unique conceptualization of each disorder (Reddit, 2017). This reaction can be explained via two important phenomena in mental health scholarship. First, individuals actively seek self-congruence by reducing discrepancies between how they see themselves and how their self-concepts are ultimately portrayed (Gonnerman et al., 2000). Second, despite psychological research focusing largely on congruence with desirable aspects of the self, mental health ailments ultimately form part of one’s self-concept as well (World Health Organization [WHO], 2001). Taken together, Allen’s series exposes a seemingly counterintuitive psychological phenomenon: Human desire for self-congruence may extend to even the most undesirable aspects of the self.

Self-congruence is increasingly being sought after, and achieved, within digital games and online virtual environments (VEs), where users’ identities are visually represented by avatars, or 3D virtual self-representations (Bailenson, 2018). As a digital incarnation of the user’s self-concept(s), an avatar (or virtual self) is composed of visual attributes that reflect parts of the user’s identity; often an idealized incarnation of the self (Jin, 2012). Because the virtual self is integral to one’s identity, self-avatar congruence has been shown to lead to psychological outcomes akin to those elicited by self-congruence in the real world. For example, avatars resembling a user’s ideal self has been shown to influence self-appraisals (Kim and Sundar, 2012). As a result, users actively manage their virtual selves to be in line with their unique identity standards and minimize undesired deviations between the self and the avatar.

To achieve self-avatar congruence, avatars are often tailored by the user to project salient visual cues (e.g., avatar height, race) related to some aspect of the self (Bailenson, 2018), a process known as customization (Kalyanaraman and Sundar, 2006). Customization contributes to self-avatar congruence by allowing customized objects (e.g., avatars) to reflect desired aspects of the user’s identity (Franke et al., 2010; Kim and Sundar, 2012). This heightened congruence, enabled via customization, has been shown to positively influence attitudes (Van Der Heide et al., 2013) and overall well-being (Fox et al., 2009).

The affordance of customization is a major draw of modern digital games, primarily because it provides users with access to ideal aspects of the self (Przybylski et al., 2012). Consequently, users seldom customize avatars, or any self-referencing content, to represent undesirable aspects of the self (Marshall, 2008; Dunn and Guadagno, 2012). Indeed, attractiveness of an avatar contributes toward the user’s connection with the character, which in turn contributes to participation in virtual communities (see Kim et al., 2012). This human proclivity for conveying the idealized self in virtual worlds, coupled with the ubiquity of customization interfaces within VEs, has relegated virtual identity research to focus on actual and desirable self-concepts (e.g., Jin, 2012; Kim and Sundar, 2012). However, given the importance of undesirable self-concepts in identity formation, emerging ethnographic research suggests there are benefits to achieving congruence with an avatar representing undesirable aspects of one’s identity.

Users of popular VEs such as Second Life (SL) are increasingly creating avatars that convey less desirable, but no less integral, self-concepts (Stewart et al., 2010). For example, Bloustien and Wood (2013, 2016) recent work identified a growing segment of SL users creating avatars that exhibit their physical disabilities (i.e., avatars representing the disabled self-concept). Where creating an avatar representing the disabled self can be beneficial to the user by fostering self-worth (see Dunn and Burcaw, 2013 for a review of disability identity), the implications of creating an avatar rooted in undesirable, mental health-related self-concepts (e.g., anxiety) remain ambiguous. Furthermore, should benefits arise from achieving congruence with an avatar imbued with undesirable self-concepts, mechanisms driving such effects are largely unknown. Thus, this paper seeks to explore the effects of imbuing avatars with a wholly undesirable, mental health-related self-concept: anxiety. Anxiety is particularly germane to this investigation due its classification as the most prevalent health-related self-concept, and a common ailment among users of VEs (Lo et al., 2005; Mehroof and Griffiths, 2010; Anxiety and Depression Association of America [ADAA], 2017).

Assuming individuals could tailor an avatar to represent an undesirable self-concept, such as the anxious self, this would present a unique conundrum in terms of affective outcomes due to competing mechanisms at play. On the one hand, the avatar may serve as an emotion-laden cue representing a negative affective state (e.g., anxiety) in its most accurate form, facilitating negative responses (i.e., anxiety thoughts) (Lilienfeld et al., 1993). Conversely, creating a more self-congruent avatar both reduces discrepancies and affords the user an increased sense of control (Marathe and Sundar, 2011), subsequently contributing to positive responses (e.g., favorable attitudes) (Carver and Scheier, 1983). These competing mechanisms pose an important theoretical query: what are the emotional and psychological effects of customizing a virtual avatar representing an individual’s anxious self-concept?

Another equally important question is whether the effects of self-avatar congruence are contingent on user-avatar interactions, or whether the affordance of mere customization suffices. Put differently, can specific interactions with a customized avatar representing an undesirable self-concept augment the effects of self-avatar congruence? Research suggests that, despite a natural inclination to reduce discrepancies with a reference value, people may also wish to engage in behaviors to distance themselves from a “feared or disliked possible self” (see Carver et al., 2000, p. 743). One such behavior may be the destruction of the avatar itself. Experiments have shown that positive evaluations of a created object dissipate upon its destruction (Norton et al., 2012). In this way, just as customization can foster congruence, destruction of a customized anxiety avatar may increase the discrepancy between one’s actual self and a negative reference value (e.g., feared or disliked possible self). Such a reappraisal, one which detaches the self from a target stimulus, has been shown to engender positive emotional responses (Kalisch et al., 2005).

The aforementioned questions ultimately emphasize the potentialities associated with customizing a virtual avatar representing one’s anxious self-concept, namely the emotional and psychological effects. To begin to address the aforementioned questions, we conducted two experiments examining avatar customization in a novel context, focusing on the affective responses to customized avatars representing the anxious self (henceforth referred to as an “anxiety avatar”). Study 1 tests whether customization of an anxiety avatar reduces negative affect (i.e., anxiety reduction) by reducing the difference between the user’s anxious self-concept and its virtual representation (i.e., discrepancy reduction). Conversely, Study 2 explores how destruction of a customized anxiety avatar may reduce negative affect by increasing the difference between the user’s anxious self-concept and its virtual representation (i.e., discrepancy enlargement).

Drawing on human–computer interaction (HCI) research and self-conceptual frameworks, this paper seeks to make several contributions. First, we address the gap in the literature by exploring self-avatar congruence in the context of undesirable self-concepts. Second, in doing so, we also assess boundary conditions of customization effects, which have largely been studied as positive consequences resulting from matching content to desirable aspects of the self. Lastly, we seek to establish the viability of avatar-based self-regulatory strategies for anxiety management, many of which can be seamlessly integrated into existing character customization interfaces across a wide swath of digital games. In the following section we examine pertinent literature, provide a review of the studies’ methodology and results, and conclude with a discussion on theoretical and practical implications.

## Literature Review

### The Virtual Self

The virtual self is conceptualized as the projection of self-concepts onto virtual avatars (Yee and Bailenson, 2007), which are graphical embodied representations of the user within VEs. Self-concepts function as a sum of a person’s beliefs and knowledge about their unique personal qualities and attributes (Mann et al., 2004), a schema storing concrete and abstract views about the “self” (Markus, 1977). Thus, the virtual self serves as “an object with certain properties” (Clegg, 2013, pp. 201–202), properties which can include both desirable and undesirable aspects of one’s identity (e.g., anxiety) (World Health Organization [WHO], 2001, p. 14). In this regard, avatars “provide access points in the creation of identity” (Taylor, 2002, p. 40), and perpetuate a consistent user identity (Peachey, 2010). Avatars also function as a rhetorical device (Kolko, 1999), with physical and psychological self-concepts outwardly communicated through salient cues embedded within the avatar (Schultze, 2010). In this way, avatars allow the user to explore different identities and project self-concepts onto avatars, transforming VEs into “social laboratories for identity study” (Peachey, 2010, p. 37).

### Self-Discrepancy Theory

The self, virtual or otherwise, is delineated across three basic domains: the actual, ought, and ideal self. The actual self is comprised of the representation of attributes that you and others believe you possess. The ought self is the representation of attributes that one believes they should possess. Lastly, the ideal self represents attributes that one would like to possess – an idealized self (see Higgins, 1987). Given the natural inclination for users to match avatars to their ideal or desirable self-concepts (Dunn and Guadagno, 2012), ideal self-concepts comprise the lion’s share of virtual identity research (e.g., Mancini and Sibilla, 2017; also see Nowak and Fox, 2018). The prominence of idealized self-representations is evident in VEs such as *World of Warcraft* (WoW), a popular massive multiplayer online role-playing game (MMORPG). In such VEs, users seldom create avatars that reflect “undesirable” aspects of the self, such as being short or obese, primarily because they do not align with the user’s idealized self (Ducheneaut et al., 2009).

Higgins (1989) self-discrepancy theory (SDT) presumes individuals actively manage (virtual) self-perceptions to be in line with their unique identity standards, a process known as self-regulation (Burke and Stets, 1999). That is, users respond to salient discrepancies between the self and a reference value (e.g., a virtual avatar) by engaging in behaviors that reduce discrepancies with the reference value (Carver and Scheier, 1990, 2004). This is important because discrepancies between and among self-concepts have been shown to relate causally to psychological well-being in experimental (Higgins et al., 1985) and longitudinal studies (Strauman, 1996), and can lead to psychological distress (Schafer et al., 1996).

### Customization and Self-Regulation

Given that humans seek congruity with their self-representations, virtual or otherwise, users may engage in discrepancy reduction within VEs by tailoring the appearance of their avatar to align with a particular self-concept. By means of a computer interface, individuals may modify the appearance of an avatar, imbuing it with desirable traits (e.g., using a tall avatar when the user is short), and aligning the virtual self with their ideal self (Bessière et al., 2007). This process of tailoring the avatar’s appearance to a desired form is known as customization (Dunn and Guadagno, 2012; Kalyanaraman and Wojdynski, 2015). As a user-initiated process, customization relies on user decisions which result in changes to, and control over, the content (Kalyanaraman and Sundar, 2006). Customization ultimately ensures that any object, virtual or otherwise, is infused with some aspect of the self (Marathe and Sundar, 2011). In doing so, research has consistently shown that customized content, be it game characters or web content, is evaluated more favorably and is ascribed greater value and importance when users are involved in its creation (see Kalyanaraman and Wojdynski, 2015).

While the effects of customization are robust, a methodological oversight continues to prevent a more nuanced understanding of how, and to what extent, matching content to the self can benefit an individual. Consider the typical experimental scenario used in any empirical investigation of customization: one group customizing a self-referencing object (e.g., an avatar) is compared with a control group who is assigned the self-referencing object. Regardless of the research question or dependent variables, the customized object is almost always tailored to reflect the ideal or actual self (e.g., Dolgov et al., 2014; Kim et al., 2015; Birk et al., 2016). This may be an experimental artifact, though its appearance is not limited to controlled lab settings, as surveys distributed across multiple VEs also reveal that a grand majority of customized avatars are rooted in an idealistic self-view (e.g., Turkay and Adinolf, 2010). That is, customization is largely studied as a process used to match content to actual or idealized aspects of the individual, overlooking other self-concepts are equally important in the construction of the self. Indeed, users have a tendency to project important aspects of one’s identity onto their avatars (e.g., Vasalou and Joinson, 2009). Yet, concentration on idealized self-concepts, particularly among communication scholars, presents a potential theoretical and empirical blind spot. While seemingly counterintuitive, matching content to undesirable aspects of the self merits investigation and may indeed already be occurring within modern VEs. Qualitative research suggests that individuals use customization interfaces to circumvent the normative tendency to build the ideal virtual self, opting instead to imbue avatars with less socially desirable self-concepts. For instance, Bloustien and Wood (2013) found that users of SL often imbue their avatars with physical disabilities as exhibited by use of virtual wheelchairs or prosthetic limbs. Ultimately, this use of avatar customization raises a unique series of questions: if humans benefit from imbuing avatars with qualities related to their physical disorders, can the same be said for mental health-related disorders? If so, is discrepancy reduction a potential mechanism driving this phenomenon?

### The Anxious Self

Despite extant identity research focusing on the three basic domains of the self, self-theorists acknowledge that the self extends beyond the actual, ought, and ideal (Gonnerman et al., 2000). For example, mental and physical health disorders can form part of an individual’s identity despite such disorders typically being viewed by the afflicted as negative or undesirable (see Larson et al., 1984). More specifically, Tregaskis (2002) articulated the “disabled self” as an encapsulation of mobility trauma within the individual, while Price et al. (1994) proposed the “depressed self” as the integration of depression into one’s identity. The present study focuses on the inclusion of anxiety into one’s self-concept (Schmukle and Egloff, 2006) and the affective implications associated with projecting this “anxious self” onto an avatar.

Anxiety is an internalized mental disorder characterized by a sense of vulnerability and tendency to respond fearfully to stressors (McNally, 1989), as well as psychological distress due to expectation of negative outcomes (Epstein, 2013). Psychologists divide anxiety into trait and state anxiety (Spielberger et al., 1970). The former describes one’s propensity to experience anxiety, whereas the latter is situational and short-term (Rule and Traver, 1983). This investigation focuses exclusively on state anxiety, as opposed to depression or other mental health ailments, for two reasons. First, state anxiety is both common and easily evoked, having been experimentally induced in a variety of psychological studies (e.g., Richards et al., 1992; D’Argembeau and Van der Linden, 2004). Second, state anxiety is transient and reactive to external factors (see Moser, 2007). Because of this, an investigation into anxiety provides high internal validity, as changes in state anxiety can be more clearly connected to experimental manipulations.

The pervasiveness of human anxiety, and its evident role in identity formation, also prompt inquiry into how depictions of one’s anxious self may influence psychological well-being. As previously mentioned, the anxious self, and other “less cherished aspects of the self” (Thoits, 2013, p. 361), form part of one’s identity (World Health Organization [WHO], 2001). SDT argues that humans seek to minimize differences between a self-concept and its reference value (an exemplar) via self-regulation. Indeed, audiences have vocalized similar desires to modify depictions of anxiety in film (Olstead, 2002) to align with their unique conceptualizations (see Hoffner and Cohen, 2012). Should the principles of SDT hold for undesirable self-concepts, a discrepancy between how one views his/her anxious self and how it is portrayed would elicit a similar urge for self-regulation.

### Anthropomorphizing Anxiety

Conveyance of psychological or emotional self-concepts onto avatars, while absent of overt cues associated with physical disabilities (e.g., wheelchairs, prosthetic limbs), can also be facilitated via customization. Specifically, the ability to assign human qualities (e.g., personality) to objects, virtual or otherwise, is defined as anthropomorphism (DiSalvo and Gemperle, 2003). Scholars in various domains have acknowledged that individuals have an anthropomorphic tendency to imbue human attributes onto non-human entities (Reeves and Nass, 1996). This anthropomorphic tendency extends to descriptions of natural phenomena (Legare et al., 2013), with humans often personifying undesirable self-concepts. For example, diseases may be referred to as “powerful monsters,” (Mukherjee, 2010, p. 45) or “emotionless aliens” (Hitchens, 2014, p. 11) by the afflicted. Similarly, cancer patients have been known to anthropomorphize their disease in a similar fashion (see Jain and Lynch, 2011). It can thus be argued that the anxious self can be anthropomorphized via avatar customization.

Contrary to the self-congruity hypothesis, which maintains that individuals seek congruence with idealized self-representations (Grubb and Grathwohl, 1967), we propose that the benefits of discrepancy reduction may extend to undesirable self-concepts (anxiety) and their exemplars (avatars) as well. Specifically, avatar customization would bring a reference value (i.e., the avatar) closer to its expected value (i.e., the disabled self-concept), a congruence which would presumably elicit positive affect (Carver and Scheier, 1986). Thus, considering the customization process, and its self-regulatory function facilitated by matching avatar appearance to aspects of the self, we propose that customizing an anxiety avatar will contribute to psychological well-being.

**H1**: Customization of an anxiety avatar will significantly reduce anxiety.

### Avatar Identification and Self-Congruence

In avatar-based VEs, self-congruence with an avatar representing one’s anxious self-concept can be thought of in terms of the degree to which the user perceives the avatar as similar to themselves. This phenomenon is known as avatar identification (Van Looy et al., 2012), and is characterized by a heightened emotional connection with the avatar (Cohen, 2001). Player-avatar identification (PAI) argues that avatar identification occurs when a user values an aspect of the avatar and perceives this aspect as important to their identity (Li et al., 2013). Hoffner and Buchanan (2005) distinguish between two distinct types of avatar identification: similarity and wishful identification. Where wishful identification is characterized by the user’s desire to be more like the avatar (Konijn and Hoorn, 2005), similarity identification is thought of as a “rapprochement between player and character” (Van Looy et al., 2012, p. 202). In this way, customizing an avatar to be more similar to one’s self-concept can thus signal self-discrepancy reduction (see Bessière et al., 2007; Kim et al., 2012).

In sum, by selecting (or modifying) physical traits of an avatar (e.g., skin color), users can imbue avatars with self-relevant concepts related to anxiety as personified by their inputs. As a result, this investigation proposes that avatars customized to represent an undesirable self-concept (i.e., the anxious self) will result in increased similarity identification with the avatar, thereby making it congruent with their anxious self-concept.

**H2**: Users who customize an anxiety avatar will exhibit significantly greater similarity identification compared to the control condition.

### Appraisal of an Anxiety Avatar

Achieving a heightened degree of avatar identification via discrepancy reduction is considered an inherently enjoyable experience contributing to positive feelings within VEs (Hefner et al., 2007). Positive feelings engendered via avatar customization are also thought to influence the user’s overall evaluation of the avatar, as avatars exhibiting greater similarities to the user contribute to positive appraisals of the avatar (i.e., attitudes) (see Nowak and Fox, 2018). Bagozzi (1992) posits that such appraisals directly influence emotional reactions. Specifically, positive or negative attitudes trigger similarly valanced affective states such that positive attitudes are associated with positive affective states, and vice versa. Furthermore, Lazarus (1991) argues that one of the three outcomes that can result as a function of appraisals are affective states (e.g., anxiety). Thus, should customization of an anxiety avatar reduce discrepancy with a relevant self-concept, this heightened similarity should in turn generate a favorable appraisal of the avatar, eliciting positive affect. In sum, the positive psychological effects of customization on the appraisal of the avatar is contingent on it being congruent with the user’s self-conceptualization of anxiety.

**H3**: The effects of customization on anxiety will be mediated by similarity identification and attitudes toward the avatar.

### Discrepancy-Enlargement

While self-regulatory feedback loops, facilitated via customization, are focused on discrepancy-reduction or “approach processes,” there are also discrepancy-enlarging loops. Discrepancy-enlarging loops, while not as commonly discussed in self-regulating feedback systems, are characterized by a purposeful deviation from a reference value. These avoidant processes are particularly relevant to this investigation given that an anxiety avatar is rooted in a “feared or disliked possible self” (see Carver et al., 2000, p. 743). Increasing the distance between a user’s feared or disliked self-concept and its reference value, namely an avatar imbued with such concepts, has also been shown to contribute to psychological well-being. Kalisch et al. (2005) found that this detachment is an optimal cognitive strategy for emotional regulation. In this way, engaging in a reappraisal of the relationship between the self and a self-representation (i.e., a customized anxiety avatar), may allow users to volitionally reduce anxiety via detachment (p. 877). The potential of avatar-based discrepancy enlargement is further bolstered by media psychology’s assumption that users largely avoid sources of distress in the first place (Vorderer and Knobloch, 2000, p. 65). Given the aforementioned discussion, we pose the following complementary research question: Can increasing discrepancy with a customized anxiety avatar reduce anxiety?

In modern VEs, a popular game mechanic that significantly, and regularly, influences the user-avatar relationship is the destruction of the avatar. Video game scholars acknowledge that the death, or destruction, of one’s avatar is an important event, one which influences psychological well-being (Wenz, 2014). For example, research has shown that individuals in *WoW* respond to the unplanned destruction of an avatar by mourning its loss in planned ceremonies (Haverinen, 2016). More specific to this investigation is the effect of avatar destruction on identification with the avatar. Nöth et al. (2008) argue that “identification of the player with the avatar breaks down in the moment of the avatar’s death” (p. 158). In this way, destruction of an avatar reduces identification, and thus represents a discrepancy-enlargement mechanism capable of influencing a user’s affective state.

**H4**: Destruction of customized anxiety avatars will reduce anxiety from pre- to post-test.

As previously mentioned, customization should engender similarity identification via the transference of relevant self-concepts onto the avatar. However, the avatar’s destruction should demarcate the self-concept with which the avatar is imbued (i.e., anxiety). That is, a distancing effect should result where the user’s desire to be like the now-distanced undesirable self-concept is significantly eroded, reducing negative affect. In this way, reduced wishful identification serves as a signal of the user’s desire to distance him/herself from the reference value. The aforementioned effects are presumably relegated to destruction of customized avatars due to any increased valuation of an object being contingent on it being successfully created by the user. Put differently, only when individuals create objects themselves can the accrued favorable valuation dissipate upon its destruction (Norton et al., 2012). In this way, destruction of a customized anxiety avatar should then result in its negative appraisal, though negative affect associated with its destruction should be offset by the positive affect elicited via discrepancy enlargement, resulting in net positive affect (i.e., anxiety reduction).

**H5**: Destruction of a customized anxiety avatar will lead to significantly less wishful identification compared to destruction of a non-customized avatar.

**H6a**: Destruction of a customized anxiety avatar will elicit positive affect (decreased anxiety) through wishful identification.

**H6b**: Destruction of a customized anxiety avatar will elicit negative affect (increased anxiety) through attitudes.

### User Control

**FIGURE 1:**
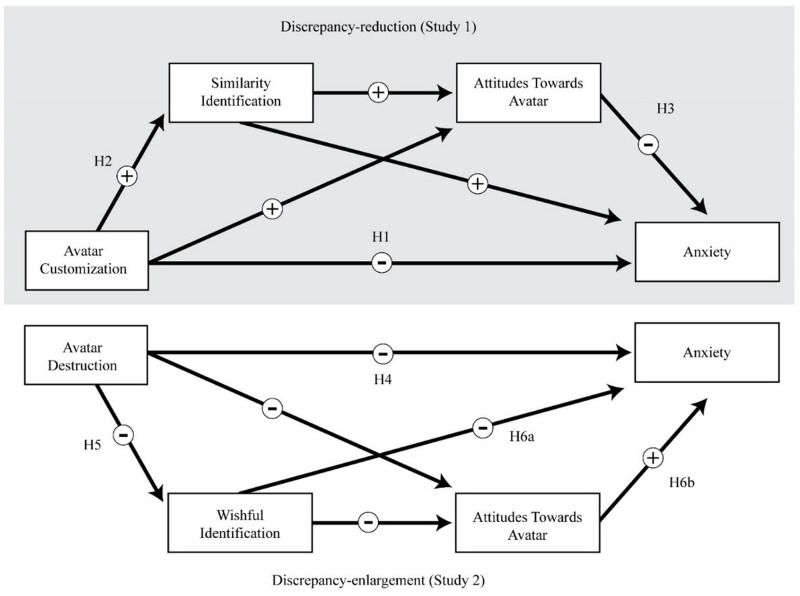
Proposed serial mediated models of anxiety reduction via discrepancy-reduction (Study 1) and discrepancy-enlargement (Study 2). ± Signs indicate predicted effect valence.

Beyond discrepancy reduction, customization can also afford users a sense of agency (or control), which bears importance due to anxiety-related distress being characterized by a “profound sense of lack of control” (Large et al., 2016, p. 199). According to the agency model of customization (Sundar, 2008), a customization interface makes the user a source, as opposed to simply a receiver, of content. This self-as-source schema allows the user to serve as the origin of the content, imbuing specific self-concepts (e.g., skin color) onto the avatar (Marathe and Sundar, 2011) while prompting feelings of control and the ability for the user to exert influence over digital content (e.g., avatars) (see Kalyanaraman and Wojdynski, 2015, p. 431). For example, in video games such as *Neverwinter Nights 2*, users can modify avatar appearance using sliders to adjust physical traits (Dunn and Guadagno, 2012). By increasing the number of dimensions that an avatar can be altered, interfaces increase perceived interactivity and a sense of user control (Kalyanaraman and Wojdynski, 2015). In this way, user control should influence affective outcomes and was thus included as a covariate in all subsequent analyses. A visualization of the proposed hypotheses is shown in Figure 1.

## Study 1

### Methods

In order to test the aforementioned hypotheses specific to avatar customization (discrepancy reduction), a 2-condition between-subjects experiment (*N* = 90) was administered through Qualtrics, with participants recruited via Amazon MTurk. The experiments in this paper were reviewed and approved by the institutional review board (IRB) and met all ethical guidelines and requirements. Participants were informed that an independent video game company was conducting a survey to examine opinions toward virtual characters. Avatar customization served as the independent variable, operationalized as the presence or absence of system features available to the user to generate a virtual creature (avatar) representing the anxious self. Dependent variables of interest included anxiety, identification with the avatar, and attitudes toward the avatar. Mean scores and standard deviations of relevant variables are shown in Table 2.

### Manipulation Check: Perceived Customization

A single 7-point Likert scale item measured participants’ level of agreement with the following statement: “The creature was personalized according to my feelings.” The item was adapted from Kalyanaraman and Sundar (2006).

### Dependent Variables

#### Anxiety

The State-trait Anxiety Inventory (STAI) measures, via self-report, the severity of current anxiety symptoms, as well as trait aspects of anxiety. The STAI served as a repeated measure, employed both prior to and immediately after the experimental conditions. The 6-item 7-point Likert scale was adapted from Marteau and Bekker (1992) (α = 0.80). Items measured the level of agreement with a variety of statements, such as “I feel tense,” and “I am worried.”

#### Identification

Self-avatar identification was delineated across two dimensions of the psychological construct: wishful and similarity identification. Wishful identification with the creature was measured via a 5-item 7-point likert scale (α = 0.73) adapted from Van Looy et al.’s (2012) scale. Items measured the level of agreement with a variety of statements related to participants’ perception of the creature, such as “I wish I could be more like the creature,” and “I imagine myself in the creature’s place.” Similarity identification was adapted from Van Looy et al. (2012) who conceptualized similarity identification as self-avatar congruence. A 5-item 7-point Likert scale (α = 0.82) assessed participants’ agreement with various statements, including “I felt connected to the creature,” and “The creature represented me as a unique individual.”

#### Attitudes

Attitudes toward the creature were measured via a 12-item 7-point Likert scale measuring user’s level of agreement with various statements including, “the creature was appealing,” and “the creature was interesting” (α = 0.96) (Kalyanaraman and Sundar, 2006).

#### User Control

The “active control” dimension of interactivity was measured via a 2-item 7-point Likert scale adapted from Liu’s (2003) factor analysis on the perceived interactivity scale (α = 0.79). Participants rated their level of agreement with the following statements, “I felt I had control over my experience,” and “My actions decided the kind of experiences I got.”

### Participants and Procedures

Among all participants, 57 identified as males and 33 identified as females, with ages ranging from 19 to 58, and an average age of 32.32 (*SD* = 7.68). After initial completion of demographic questions, participants were instructed to reflect for 1 min on a time when they experienced a high level of anxiety. This anxiety manipulation was deemed appropriate given that past representations of events elicit similar feelings during the re-experience (see D’Argembeau and Van der Linden, 2004). Furthermore, the cognitive appraisal of anxiety-related events has been shown to cause anxiety (Lilienfeld et al., 1993). After the reflection, self-reported anxiety was measured (pretest scores). Participants were then randomly assigned to either the customization or control condition.

**FIGURE 2:**
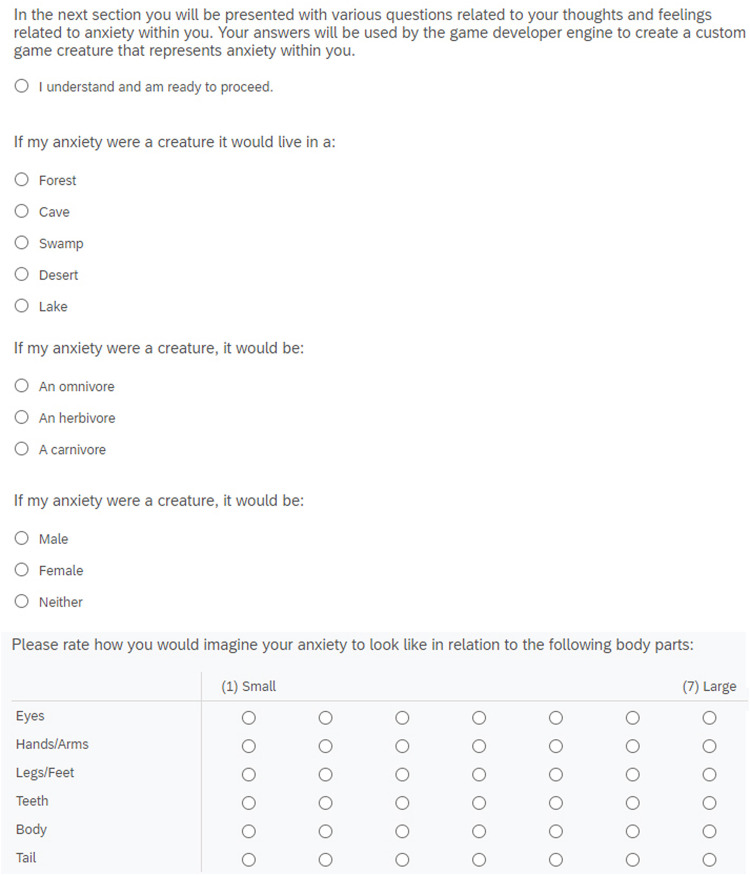
Avatar customization interface in studies 1 and 2.

**FIGURE 3:**
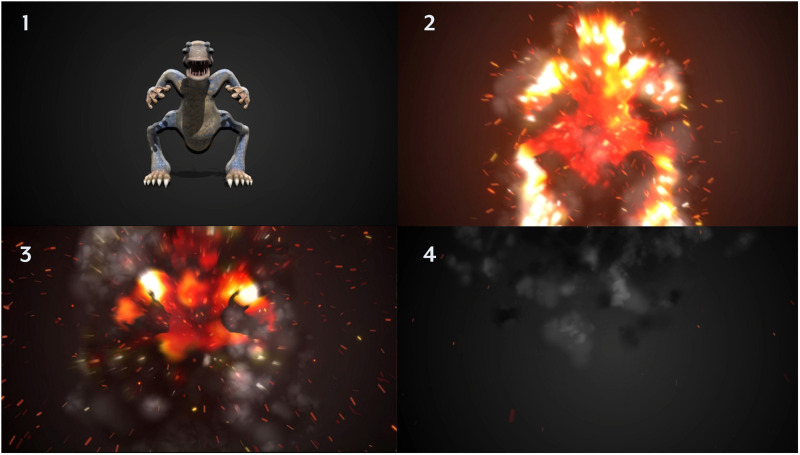
Visualization of the avatar destruction animation sequence used in study 2.

In the customization condition, participants were prompted to select from a range of parameters they felt best represented anxiety within themselves (see Figure 2). Specifically, participants were presented with nine statements about the creature, including “If my anxiety were a creature it would live in a desert/swamp/cave,” and “If my anxiety were a creature it would be an omnivore/herbivore/carnivore.” The statements were selectable within Qualtrics via a series of customized radio buttons, each corresponding to the aforementioned statements. After selecting the items which best represented anxiety within themselves, a screen instructed participants to wait for the system to generate a 3D creature representative of their anxiety (see Figure 3). A single, randomized creature was generated prior to the experiment using the custom creature creator within the video game *SPORE* and exported as a 3D model using Adobe Photoshop. This ensured that all participants were shown the same creature regardless of their chosen customization parameters. In the control condition, these questions were bypassed, and participants were presented with the same creature along with a prompt stating that the creature was generated by the system to represent anxiety within them. Upon exposure to the creature, both groups were then asked to examine the creature for 30 s, after which their self-reported anxiety levels were measured again (posttest scores). Lastly, participants completed a thought-listing exercise and finished the remainder of the survey.

## Results

### Manipulation Check: Perceived Customization

A one-way ANOVA revealed that the customization manipulation was successful, such that there were significant differences in perceived customization between the customization group (*M* = 4.71, *SD* = 1.44) and the control group (*M* = 2.8, *SD* = 1.6), *F*(1,87) = 35.26, *p* < 0.0001. Correlations among all of the variables are shown in Table 1. Mean scores and standard deviations of relevant variables are shown in Table 2.

**TABLE 1:** Pearson correlation matrix for all dependent variables in Study 1.

	Perceived Customization	Similarity Identification	Wishful Identification	Attitudes	User Control	STAI (T1)	STAI (T2)
Perceived Customization	–	0.71**	0.5**	0.41**	0.24*	0.12	0.04
Similarity Identification		–	0.81**	0.58**	0.21*	0.13	0.06
Wishful Identification			–	0.61**	0.15	0.07	–0.01
Attitudes				–	0.38**	–0.11	0.41**
User Control					–	–0.18	–0.33**
STAI (T1)						–	0.81**
STAI (T2)							–

*p < 0.05, **p < 0.01.

**TABLE 2:** Study 1 mean scores and standard deviations across experimental conditions.

	No Customization	Customization
Perceived Customization	2.8 (1.6)	4.71 (1.44)
State Anxiety T1	18.51 (7.37)	19.35 (7.19)
State Anxiety T2	20.06 (7.29)	17.68 (7.32)
Wishful Identification	2.12 (1.36)	2.85 (1.79)
Similarity Identification	2.5 (1.42)	3.73 (1.55)
Attitudes	3.26 (1.67)	3.74 (1.71)
User Control	4.76 (1.59)	4.95 (1.37)

*Standard deviation values are shown in parenthesis.*

### Dependent Variables

#### Anxiety

A repeated-measures analysis of variance (rANOVA) was run with customization as an independent factor, and STAI at pretest and posttest as the dependent within-subjects factor. Results showed no differences in anxiety at pretest between groups [*F*(1,88) = 0.302, *p* > 0.05, $\eta_{\text{p}}^{2}$ = 0.003]. However, there was a significant interaction between customization and anxiety change from pretest to posttest [*F*(1,88) = 13.15, *p* < 0.001, $\eta_{\text{p}}^{2}$ = 0.13]. That is, there was a significant increase in anxiety from pretest (*M* = 18.51, *SD* = 7.37) to posttest (*M* = 20.06, *SD* = 7.29) among those in the control condition (*M*_*difference*_ = −1.55, *p* = 0.01; 95% CI [−2.8, −0.31], $\eta_{\text{p}}^{2}$ = 0.06), supporting the efficacy of the anxiety manipulation. Conversely, anxiety from pretest (*M* = 19.35, *SD* = 7.19) to posttest (*M* = 17.68, *SD* = 7.32) significantly decreased for those in the customization condition (*M*_*difference*_ = 1.66, *p* < 0.01; (95% CI [0.418, 2.92]), $\eta_{\text{p}}^{2}$ = 0.07). Thus, H1 was supported.

#### Wishful Identification

A one-way ANOVA revealed that customization significantly influenced wishful identification with the avatar such that customized avatars were more wishfully identified with (*M* = 2.85, *SD* = 1.79) compared to those in the control group (*M* = 2.12, *SD* = 1.36), *t*(88) = −2.19, *p* < 0.05, $\eta_{\text{p}}^{2}$ = 0.05.

#### Similarity Identification

A one-way ANOVA revealed that customization led to significantly greater levels of similarity identification with the avatar (*M* = 3.73, *SD* = 1.55) compared to the control group (*M* = 2.5, *SD* = 1.42), *t*(88) = −3.91, *p* < 0.001, $\eta_{\text{p}}^{2}$ = 0.14. Thus, customization led to greater levels of self-avatar congruence, supporting H2.

#### Attitudes

A one-way ANOVA revealed no significant differences in attitudes toward the avatars between participants in the customization (*M* = 3.74, *SD* = 1.71) and control group (*M* = 3.26, *SD* = 1.67) *t*(88) = −1.35, *p* > 0.05, $\eta_{\text{p}}^{2}$ = 0.02.

#### User Control

A one-way ANOVA revealed that avatar customization did not lead to significantly greater levels of user control (*M* = 4.95, *SD* = 1.37) compared to the control condition (*M* = 4.76, *SD* = 1.59) *t*(88) = −0.6, *p* > 0.05, $\eta_{\text{p}}^{2}$ = 0.004. Correlations among all of the variables are shown in Table 1.

### Serial Mediation

To test the hypothesis that customization of an anxiety avatar can reduce anxiety through similarity identification and attitudes (H3), a bootstrapped (5,000 resamples) serial mediation analysis (Hayes, 2015, p. 10) was conducted. The PROCESS macro (Model 6; Hayes, 2012) was used to determine whether customization predicted similarity identification, and whether similarity identification influenced attitudes toward the creature, which subsequently influenced participants’ anxiety at posttest.

**FIGURE 4:**
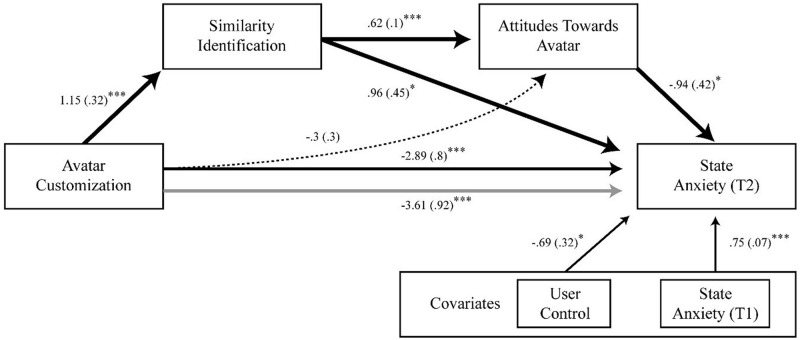
Study 1 coefficients for serial mediation analysis of avatar customization on anxiety through proposed mediators. Bold paths are statistically significant, while dashed paths are non-significant. Standard error terms are shown in parentheses. Grey line denotes the effect of avatar customization on anxiety when mediators are included in the model. *p < 0.05, **p < 0.01, ***p < 0.001.

The mediation model (Figure 4) established customization as a significant predictor of similarity identification with the avatar (*b* = 1.15, *p* < 0.01; 95% CI [0.51, 1.8]). Similarity identification subsequently predicted attitudes toward the avatar (*b* = 0.62, *p* < 0.01; 95% CI [0.41, 0.83]). Lastly, attitudes were significantly inversely related to state anxiety at posttest (*b* = −0.94, *p* < 0.05; 95% CI [−1.8, −0.09]). The model demonstrated significant indirect effects of customization on anxiety through similarity identification and attitudes, resulting in decreased anxiety (*b* = −0.68, bootstrapped *SE* = 0.32; 95% CI [−1.62, −0.21]). However, the inclusion of mediators increased the predictive utility of customization on anxiety at posttest, leading to anxiety reduction (*b* = −3.61, *p* < 0.01; 95% CI [−5.45, −1.77]), larger than the total direct effect (*b* = −2.89, *p* < 0.01; 95% CI [−4.48, −1.3]). A discussion on the nature of this effect is addressed in the general discussion section.

## Discussion: Study 1

Study 1 tested the assumption that customization of an anxiety avatar would reduce anxiety through increased similarity identification (discrepancy reduction) and attitudes. The results support the proposed relationship between customization and similarity identification. Mere affordance of customization increased perceived congruence between the user’s self-concept (i.e., the anxious self) and the customized avatar representing that self-concept. Moreover, this discrepancy reduction led to favorable evaluation of the avatar despite representing an undesirable aspect of the user (Figure 4), ultimately reducing anxiety at posttest.

The theoretical rationale behind this finding is rooted in the need for consistency between and among our self-schemas, a proposition that Study 1 extends to undesirable self-concepts. However, there are two important caveats that must be considered when examining congruity with the anxious self, and perhaps other undesirable self-concepts. First, studies examining congruence between and among self-concepts acknowledge that such concepts are largely positive (Aaker, 1999). This is not the case with the anxious self, a schema comprised of wholly undesirable traits. Second, a primary motivation behind achieving “self-congruity” is to aid in self-presentation. While participants ultimately created a representation of the anxious self, the avatar was not used in a social context. Thus, while self-consistency may explain the emotional benefits of customizing an anxiety avatar, further research is needed to understand what makes congruence with undesirable self-concepts, as opposed to desirable ones, theoretically different. Overall, these findings suggest that customization of the anxious self can elicit positive affect via discrepancy reduction.

## Study 2

### Method

**FIGURE 5:**
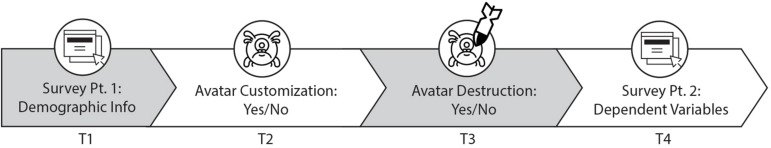
Visualization of the experimental procedures for study 2.

Where Study 1 argued that customization of the anxious self would elicit positive affect via discrepancy reduction, Study 2 posits that discrepancy enlargement, by way of destroying the avatar, may also reduce anxiety (Figure 1). To explore this alternative mechanism, a 2 (customization: yes, no) × 2 (destruction: yes, no) between-subjects (*N* = 122) experimental design was employed using Amazon MTurk. Participants were instructed to destroy an avatar representing anxiety within themselves after its creation (see Figure 5). The same variables from Study 1 were examined. There was a total of 65 male and 57 female participants ranging from 21 and 70 years old (*M* = 33.8, *SD* = 9.5).

Study 2 employed the same experimental design as Study 1, with one exception: after participants were shown the anxiety avatar (customized or non-customized), participants in the destruction condition were instructed to initiate an animation which depicted the avatar being destroyed in a disintegration animation. The 15-s animation was created using Adobe Premiere Pro and Adobe After Effects and was embedded within the survey. Participants in the non-destruction condition also watched an animation, though it instead showed the creature in an idle pose. After exposure, self-assessed anxiety was measured (posttest), followed by a thought-listing exercise, and the remaining dependent measures.

## Results

### Manipulation Check: Perceived Customization

Mean scores and standard deviations of relevant variables are shown in Table 3. All Cronbach alpha scores were within the accepted range. The manipulation check of customization proved successful such that participants in the customization condition perceived a greater level of customization (*M* = 4.37, *SD* = 1.81) compared to those who did not (*M* = 3.63, *SD* = 1.93) [*F*(1,118) = 4.61, *p* < 0.05, $\eta_{\text{p}}^{2}$ = 0.038].

**TABLE 3:** Study 2 mean scores and standard deviations across experimental conditions.

	No Destruction/No Customization	Destruction/No Customization	No Destruction/Customization	Destruction/Customization
Perceived Customization	3.58 (2.09)	3.68 (1.77)	4.33 (1.98)	4.41 (1.66)
Similarity Identification	3.52 (1.97)	3.36 (1.72)	3.93 (1.69)	3.5 (1.61)
Wishful Identification	3.09 (2.09)	2.89 (1.97)	3.22 (1.89)	1.83 (1.2)
Attitudes	3.64 (1.62)	4.03 (1.6)	3.93 (1.37)	3.32 (1.48)
User Control	4 (1.69)	4.22 (1.49)	4.51 (1.61)	4.78 (1.35)
Anxiety Change	−0.23 (0.91)	0.13 (0.62)	−0.26 (1.22)	0.35 (1.03)

*Standard deviation values are shown in parenthesis. Anxiety change is the standardized *Z*-score difference in state anxiety from pretest to posttest. Negative values imply negative affective response (i.e., increase in anxiety), whereas a positive value indicates positive affect (i.e., decrease in anxiety).*

### Dependent Variables

#### Anxiety

As predicted, a two-way repeated measures analysis of variance (rANOVA) running avatar customization and avatar destruction as independent factors revealed a significant decrease in anxiety from pretest to posttest among those who destroyed a customized anxiety avatar [*F*(1,118) = 12.94, *p* < 0.0001, $\eta_{\text{p}}^{2}$ = 0.10]. Thus, H4 was supported. Similarly, while not predicted, destruction significantly reduced anxiety from pretest to posttest among participants who did not customize the avatar [*F*(1,118) = 5.03, *p* < 0.05, $\eta_{\text{p}}^{2}$ = 0.04].

#### Wishful Identification

With regards to H5, there was a significant main effect of avatar destruction on wishful identification [*F*(1,118) = 5.82, *p* = 0.01, $\eta_{\text{p}}^{2}$ = 0.04]. Furthermore, pairwise comparisons revealed destruction of a customized avatar elicited significantly less wishful identification (*M* = 1.83, *SD* = 1.2) compared to the non-destruction group (*M* = 3.22, *SD* = 1.89) [*F*(1,118) = 9.05, *p* < 0.01, *$\eta_{\text{p}}^{2}$* = 0.07]. Thus, H5 was supported.

### Serial Mediation

To test whether destruction of a customized avatar reduced anxiety through wishful identification, a bootstrapped (5,000 resamples) serial mediation analysis was conducted using the PROCESS macro (Model 6; Hayes, 2012). This model was chosen to examine the direct and indirect effects of the proposed variables, and spotlighted participants in the customization conditions (*N* = 62). Avatar destruction served as the independent variable, with wishful identification and attitudes included as mediators, and posttest anxiety as the dependent variable.

The results of the bootstrapped serial mediation analysis revealed that wishful identification and attitudes mediated the relationship between avatar destruction and anxiety. The indirect effects of avatar destruction through solely wishful identification (Ind1) resulted in positive affect (decreased anxiety) (*b* = −3.39, boostrapped *SE* = 1.3; 95% CI [−6.64, −1.35]), in support of H6a. Conversely, the indirect effects through wishful identification and attitudes (Ind2) resulted in negative affect (increased anxiety) (*b* = 1.4, bootsrapped *SE* = 0.75; 95% CI [0.31, 3.55]), in support of H6b. Inclusion of wishful identification and attitudes into the model rendered the direct effect of destruction on anxiety non-significant (*b* = −1.17, *p* > 0.05, 95% CI [−4.59, 2.24]), indicative of mediation. Furthermore, this indirect effect of destruction on anxiety through wishful identification was significantly different than zero, as shown by the significant Sobel test statistic (z = −2.22, *p* < 0.05), supporting the proposed discrepancy-enlargement mechanism.

## Discussion: Study 2

**FIGURE 6:**
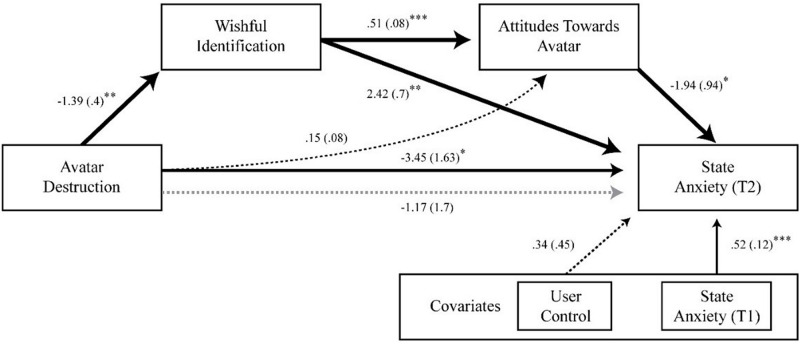
Path coefficients for serial mediation analysis of avatar destruction on anxiety through wishful identification and attitudes. Bold paths are statistically significant, while dashed paths are non-significant. Standard errors are shown in parentheses. Grey line denotes the effect of avatar destruction on anxiety when mediators are omitted from the model. *p < 0.05, **p < 0.01, ***p < 0.001.

Study 2 extended beyond mere customization, exploring how destruction of an anxiety avatar can reduce anxiety via discrepancy enlargement. The results show that the destruction of an anxiety avatar reduces user-avatar identification, subsequently reducing state anxiety. Destruction also significantly influenced appraisal of the avatar through reduction of wishful identification, indicative of discrepancy enlargement. In sum, customization aided in discrepancy reduction, while subsequent destruction initiated a discrepancy-enlargement loop, distancing the user from the undesirable self-representation, eliciting positive affect via this detachment (see Figure 6).

## General Discussion

At the crux of both studies is the notion that content which is tailored to reflect some aspect of the self can influence psychological well-being. Results across both experiments found that exposure to avatars customized to represent the user’s conceptualization of their anxiety translated into a significant reduction in state anxiety. By increasing similarity identification (Study 1: discrepancy reduction) or decreasing wishful identification with the avatar (Study 2: discrepancy enlargement), customized anxiety avatars proved to significantly influence psychological well-being via self-regulation. In sum, satisfying the drive for self-regulation by tailoring an avatar to match a particular aspect of the self contributes to psychological well-being even when the self-concept in question is wholly undesirable.

### Anxiety Begets Anxiety

As it stands, the findings contest a long-held belief in anxiety research: anxiety begets anxiety (see Corr, 2011). The adage proposes a cycle wherein exposure to anxiety-related stimuli triggers anxiety thoughts, which exacerbate anxious feelings. However, if this were the case, viewing an avatar representing the user’s anxiety would only serve to intensify anxiety, though this was not the case when the avatars were customized in Study 1. Indeed, after the anxiety induction exercise, participants in the control conditions experienced an increase in anxiety when presented with the non-customized anxiety avatar. Yet, participants who customized the anxiety avatar experienced a reduction in anxiety despite the avatar being considered a more accurate representation of their negative emotional state; a visual which would, in theory, propagate anxious feelings.

The proposed mechanism driving this effect is the ability for customization to reduce discrepancies between the user and a self-relevant object (avatar). However, an alternative explanation may also be explained by the fact that customization is inherently an enjoyable experience (Schnurr and Scholl-Grissemann, 2015). That is, the mere process of creation may have afforded sufficient positive affect to counteract any anxiety thoughts. In theory, Study 2 should have replicated these results, yet our results found the reverse to be true: those who customized, but did not destroy, their anxiety avatar experienced an increase in anxiety. A potential explanation may be rooted in subtle differences in the presentation of the customized avatars across both studies. Whereas Study 1 presented the avatars as static, 3D models, Study 2 presented the avatars in an animated 15-s video clip with sound effects (see Figure 3). Considering that dynamic (animated) visuals yield stronger influences over human emotions and psychological involvement (e.g., Wu et al., 2014), it may be that exposure to a dynamic anxiety avatar influenced emotional responses in ways that offset the benefits accrued through discrepancy reduction. Future investigations should explore these possibilities (i.e., mere customization, dynamic animation) to determine whether self-regulation is indeed the primary mechanism driving anxiety reduction.

### Expanding the Scope of Customization

The results also expand the scope of customization research, which has almost exclusively focused on matching content, whether avatars or clothes, to desirable aspects of our identity (e.g., Kaiser et al., 2017; Mancini et al., 2019). Within this contemporary customization paradigm, individuals are presumed to customize avatars to match their idealized selves, or tailor clothes to exhibit desirable personality traits (e.g., adventurous). Indeed, a survey of MMORPG gamers and their avatars uncovered four primary avatar types, or discrepancy profiles, of which three involved avatars created to convey idealized and desirable features different than the user’s offline self (Mancini and Sibilla, 2017).

While it may not be intuitive to allow users to customize content to reflect unappealing facets of their identity, such as the anxious self, it is evident that there are implications associated with doing so. Indeed, from a phenomenological perspective, demonstrating the positive effects of matching content to undesirable self-concepts bolsters SDT’s central argument, showing that self-regulation also applies to aspects of our identity that are not housed within the three main selves. Under what other circumstances this form customization can render psychological benefits remains to be explored, however.

Given the ubiquity of customization among online retailers, matching content to undesirable aspects of the self may indeed be applicable, and appropriate, in industries such as fashion. Consider the Saks Fifth Avenue’s “The Future Is Stigma Free” clothing line, which promotes shirts designed to reduce discrimination surrounding mental health illness. Though the shirts feature the titular phrase, they do not specifically mention or depict stigmatized aspects of the self, such as depression or anxiety. In this case, allowing users to customize the shirts to depict their mental illness, whether through color or imagery modification, can lead to several benefits. This form of customization would theoretically contribute to favorable evaluations of the product (Franke et al., 2010). Additionally, given the results, customization of such an “anxiety shirt” would also contribute to an improved emotional state, despite representing the illness in question.

### Destruction as Self-Distancing

Having established the theoretical and phenomenological value of customizing an avatar representing the anxious self, Study 2 expands our understanding of how customization’s impact on psychological well-being can be enhanced through subsequent interactions with the avatar. Results from our follow-up study found interactions that increase, rather than decrease, user-avatar discrepancies can also reduce anxiety. Destruction, an act shown to reverse any favorable effects garnered by creating an object (Norton et al., 2012), was used as this distancing interaction. Destroying a customized anxiety avatar significantly reduced anxiety, presumably by psychologically distancing the user from the source of distress. This provides further empirical support for self-regulation strategies, and novel insight into the psychological effects of destruction. Additionally, it raises questions about how user control can be conceptualized in VEs. Because destruction did not lead to a significantly higher sense of user control, it remains to be seen whether other forms of control, such as controlling the size or movement of the avatar, could facilitate anxiety reduction without discrepancy-enlargement. Future research should explore the various manifestations of user control as it pertains to the user-avatar dynamic, and its influence on psychological well-being.

Through a developmental prism, the findings reinforce the notion that customization of a self-relevant avatar can clarify or modify one’s self-concept(s), in turn shaping one’s identity (Schlenker, 1985, p. 66). Combined with the understanding that our identities are inextricably linked to our psychological well-being, it is reasonable to propose avatar customization as a promising anxiety management strategy within digital games and e-mental health. Hypothetically, regular interactions with a customized anxiety avatar may be beneficial if they reinforce discrepancy-enlargement loops, facilitating continual anxiety reduction. However, several aspects of this form of self-regulation remain unclear. As previously alluded to, while this study explored destruction as a distancing mechanic, there are other forms of interactions which may have a different self-regulating effect. For example, distance may be operationalized as the degree of a power disparity between the user and the anxiety avatar. Considering that psychological research has established an association between a sense of power and height (Duguid and Goncalo, 2012), manipulating the avatar be smaller than the user may facilitate a similar distancing mechanism, or a shift in the power dynamic. This action would also presumably increase the user’s sense of control, which would contribute to anxiety reduction. Regardless of the nature of the interaction, it is unclear what affective outcomes arise as a result of extended exposure to, or interaction with, an anxiety avatar.

## Limitations and Future Research

Worth noting are issues related to the efficacy of the experimental manipulation given individual differences related to anxiety and depression. According to Dobson (1986), self-schema theory argues that people with depression, for example, generalize and interpret their undesirable self-concept differently from non-depressives (p. 191). MacLeod and Mathews (1988) also acknowledged parsing between state and trait anxiety as being problematic. Their study found that, among those with high trait anxiety, exposure to threat cues focused attention to the stimulus, whereas increased state anxiety led to avoidance among those with low trait anxiety. Furthermore, studies have also shown that trait anxiety is associated with selective processing, which is elicited by state anxiety (Williams et al., 1988). Lastly, the studies could be strengthened by exploring the role of trait anxiety in shaping affective outcomes associated with customizing (and destroying) an anxiety avatar.

Another important note relates to the indirect effects found in Study 1. While significant, similarity identification and attitudes did not fully mediate the direct effect of customization on anxiety at posttest. These findings signal an absence of full mediation and expose a significant suppressor effect. Put differently, inclusion of similarity identification and attitudes enhanced the predictive capability of the independent variable (customization). While this finding diminishes the predictive power of the proposed discrepancy-reduction mechanism at-play, it does raise important questions regarding individual differences (e.g., locus of control), and what other mediating psychological variables pertinent to anxiety research (e.g., self-efficacy) may need to be accounted for.

One explanation may relate to participants’ locus of control, or beliefs about the degree to which one’s control of outcomes result from internal or external factors (Rotter, 1966). Individual differences in locus of control can influence affective and behavioral outcomes (see Deci and Ryan, 2000). Considering the avatar was not customized in real-time (i.e., users did not actively change the morphology of the 3D model through their mouse inputs), individual differences in locus of control may have influenced outcomes. Participants with internal loci of control may have felt they exerted insufficient influence over the avatar’s appearance, reacting negatively upon its presentation, whereas those with external loci of control might have attributed discrepancies to the customization interface itself. While locus of control is conceptualized as a trait variable, technological affordances can shift perceived locus of control (Ahn et al., 2014) in the short term, eliciting downstream effects. As noted earlier, Marathe and Sundar (2011) acknowledge that customizable interfaces “place the locus of control within the user” (p. 732). Thus, different measures of “user control” (e.g., Waddell et al., 2015) may benefit future studies.

Lastly, there are two important caveats regarding the avatar customization interface employed in this study which limit the ecological validity of the results. First, the customization manipulation employed in the studies were rudimentary and not comparable to character creation interfaces common in modern video games. Whereas modern games allow a greater bandwidth of editable physical features on an avatar, the current investigation used text-based radio buttons to provide the user with control over limited physical parameters on their avatar. For example, users could express their desired tail length but not the specific color or skin texture. Despite the limited malleability of the avatar, the manipulation of customization abides by the fundamental principle of customization: a particular output can be altered so as to match desired attributes dictated by the user’s inputs. Second, users did not control the avatar in a gaming context at any point. This is particularly important considering that avatars are typically under the “player’s control” (Kromand, 2007). Despite the anxiety avatars existing in isolation outside of the direct influence of the user upon their creation, the study did successfully create the illusion of customization, and thereby fostered the necessary conditions to connect users to their graphical representation (i.e., increased identification) despite not using the avatar in a game scenario. Indeed, our results suggest that avatar creation alone may yield meaningful outcomes for users.

## Conclusion

The proposition that increasing similarity identification can function as discrepancy reduction (Study 1), and reduction in wishful identification can function as discrepancy enlargement (Study 2), was largely supported across two experiments. Through a simple customization interface, participants were able to imbue avatars with salient characteristics indicative of their unique conceptualization of anxiety within themselves. As a result, users achieved congruence with the anthropomorphized depiction of the anxious self. That is, how they perceived anxiety within themselves, and how it was represented, became more aligned. In our sample, this elicited positive affect (reduced anxiety) via improved appraisal of the avatar. Similarly, decreasing identification with the avatar, through destruction, reduced anxiety as well. While customization is “self-as-source,” it is our hope that our findings direct attention toward other aspects of the self, and the psychological implications of doing so. From an applied perspective, there are multiple avenues through which to apply this knowledge. Consider modern digital games; seldom are players afforded the opportunity to customize in-game enemy characters. Given the potential benefits of creating and destroying anxiety avatars, game designers may consider implementing such mechanisms to allow players to destroy self-relevant characters in ways that contribute to their well-being.

Interactions with, and as, an anxiety avatar may be also be augmented by the unique affordances of emerging media platforms, such as augmented reality (AR) and virtual reality (VR). Both AR and VR allow the user to modify their appearance (self-representation) by overlaying virtual content over their body or by granting them control over a virtual avatar in a VE accessed via a head-mounted display, respectively (see Bailenson, 2018). In the latter case, users are afforded the capacity for “avatar embodiment,” which describes the way in which VR systems swap (part of) a user’s body with a virtual proxy (e.g., Spanlang et al., 2014). This illusion of inhabiting another virtual body is also referred to as “body transfer” (Bailey et al., 2016) or a “body ownership illusion” (Slater, 2009) and has been shown to have wide-ranging effects. For example, characteristics of an avatar embodied by a user have been shown to influence motivation (Hudson and Hurter, 2016), cognitive task performance (Chang et al., 2019), and, more pertinent to this investigation, anxiety and fear (Ferrer-Garcia et al., 2017). Acknowledging the influence of avatar embodiment through XR platforms, future work should investigate the implications of creating and embodying anxiety avatars, either through AR filters akin to what is found on apps like Snapchat, or through avatar embodiment in VR. For example, the recent game *Hellblade: Senua’s Sacrifice* leverages VR to allow users to embody a character suffering from mental illness. The implications of such embodied experiences are largely unknown, though such trends underscore the relationship between avatar design and mental health in video games.

In sum, there are clear theoretical and practical implications associated with customizing virtual avatars representing one’s anxiety, and the effects are only beginning to be understood. Yet, it is evident that avatar-based e-health solutions warrant further attention, and placement alongside recent HCI developments for health and wellbeing as noted by Blandford (2019).

## Data Availability Statement

The raw data supporting the conclusions of this article will be made available by the authors, without undue reservation.

## Ethics Statement

The studies involving human participants were reviewed and approved by The University of Florida Institutional Review Boards (IRBs). The patients/participants provided their written informed consent to participate in this study.

## Author Contributions

DP contributed to the experimental design, data collection, stimulus development, analysis, and manuscript writing. SK contributed to the experimental design, analysis, and manuscript writing. Both the authors contributed to the article and approved the submitted version.

## Conflict of Interest

The authors declare that the research was conducted in the absence of any commercial or financial relationships that could be construed as a potential conflict of interest.

## References

[B1] Aaker J. L. (1999). The malleable self: the role of self-expression in persuasion. *J. Mar. Res.* 36 45–57. 10.2307/3151914

[B2] Ahn S. J. G. Bailenson J. N. Park D. (2014). Short-and long-term effects of embodied experiences in immersive virtual environments on environmental locus of control and behavior. *Comp. Hum. Beh.* 39 235–245. 10.1016/j.chb.2014.07.025

[B3] Anxiety and Depression Association of America [ADAA] (2017). *Generalized Anxiety Disorder (GAD).* Available online at: https://adaa.org/understanding-anxiety/generalized-anxiety-disorder-gad (accessed November 20, 2019).

[B4] Bagozzi R. P. (1992). The self-regulation of attitudes, intentions, and behavior. *Soc. Psychol. Q.* 55 178–204. 10.2307/2786945

[B5] Bailenson J. (2018). *Experience on Demand: What Virtual Reality Is, How It Works, and What It Can Do.* New York, NY: W. W. Norton.

[B6] Bailey J. O. Bailenson J. N. Casasanto D. (2016). When does virtual embodiment change our minds? *Presence Teleoper. Virtual Environ.* 25 222–233. 10.1162/PRES_a_00263

[B7] Bessière K. Seay A. F. Kiesler S. (2007). The ideal elf: identity exploration in World of Warcraft. *Cyberpsychol. Behav.* 10 530–535. 10.1089/cpb.2007.9994 17711361

[B8] Birk M. V. Atkins C. Bowey J. T. Mandryk R. L. (2016). “Fostering intrinsic motivation through avatar identification in digital games,” in *Proceedings of the 2016 CHI Conference on Human Factors in Computing Systems*, (San Jose, CA), 2982–2995.

[B9] Blandford A. (2019). HCI for health and wellbeing: challenges and opportunities. *Int. J. Human Comput. Stud.* 131 41–51. 10.1016/j.ijhcs.2019.06.007

[B10] Bloustien G. Wood D. (2016). Visualising disability and activism in Second Life. *Curr. Sociol.* 64 101–121. 10.1177/0011392115596025

[B11] Bloustien G. F. Wood D. (2013). Face, authenticity, transformations and aesthetics in Second Life. *Body Soc.* 19 52–81. 10.1177/1357034X12462250

[B12] Burke P. J. Stets J. E. (1999). Trust and commitment through self-verification. *Soc. Psychol. Q.* 64 347–366. 10.2307/2695833

[B13] Carver C. S. Scheier M. F. (1983). A control-theory approach to human behavior, and implications for problems in self-management. *Adv. Cogn. Behav. Res. Ther.* 2 127–194. 10.1016/b978-0-12-010602-8.50010-9

[B14] Carver C. S. Scheier M. F. (1986). “Functional and dysfunctional responses to anxiety: the interaction between expectancies and self-focused attention,” in *Self-Related Cognitions in Anxiety and Motivation*, ed. Schwarzer R. (Abingdon-on-Thames: Taylor & Francis Group), 111–141.

[B15] Carver C. S. Scheier M. F. (1990). Origins and functions of positive and negative affect: a control-process view. *Psychol. Rev.* 97:19 10.1037/0033-295X.97.1.19

[B16] Carver C. S. Scheier M. F. (2004). “Self-regulation of action and affect,” in *Handbook of Self-Regulation: Research, Theory, and Applications*, eds Vohs K. D. Baumeister R. F. (New York, NY: Guilford Press), 13–39.

[B17] Carver C. S. Sutton S. K. Scheier M. F. (2000). Action, emotion, and personality: emerging conceptual integration. *Pers. Soc. Psychol. Bull.* 26 741–751. 10.1177/0146167200268008

[B18] Chang F. Luo M. Walton G. Aguilar L. Bailenson J. (2019). Stereotype threat in virtual learning environments: effects of avatar gender and sexist behavior on women’s math learning outcomes. *Cyberpsychol. Behav. Soc. Netw.* 22 634–640. 10.1089/cyber.2019.0106 31580726

[B19] Clegg J. W. (2013). *Self-Observation in the Social Sciences.* New Brunswick, NJ: Transaction Publishers.

[B20] Cohen J. (2001). Defining identification: a theoretical look at the identification of audiences with media characters. *Mass Comm. Soc.* 4 245–264. 10.1207/S15327825MCS0403_01

[B21] Corr P. J. (2011). Anxiety: Splitting the phenomenological atom. *Pers. Individ. Dif.* 50, 889–897. 10.1016/j.paid.2010.09.013

[B22] D’Argembeau A. Van der Linden M. (2004). Phenomenal characteristics associated with projecting oneself back into the past and forward into the future: influence of valence and temporal distance. *Conscious. Cogn.* 13 844–858. 10.1016/j.concog.2004.07.007 15522635

[B23] Deci E. L. Ryan R. M. (2000). The” what” and” why” of goal pursuits: human needs and the self-determination of behavior. *Psychol. Inq.* 11 227–268. 10.1207/S15327965PLI1104_01

[B24] DiSalvo C. Gemperle F. (2003). “From seduction to fulfillment: the use of anthropomorphic form in design,” in *Proceedings of the 2003 International Conference on Designing Pleasurable Products and Interfaces*, (Pittsburgh, PA), 67–72. 10.1145/782896.782913

[B25] Dobson K. S. (1986). “The self-schema in depression,” in *Perception of Self In Emotional Disorder and Psychotherapy*, eds Blankstein K. R. Hartman L. M. (New York, NY: Springer), 187–217. 10.1007/978-1-4613-1793-7_8

[B26] Dolgov I. Graves W. J. Nearents M. R. Schwark J. D. Volkman C. B. (2014). Effects of cooperative gaming and avatar customization on subsequent spontaneous helping behavior. *Comp. Human Behav.* 33 49–55. 10.1016/j.chb.2013.12.028

[B27] Ducheneaut N. Wen M. H. Yee N. Wadley G. (2009). “Body and mind: a study of avatar personalization in three virtual worlds,” in *Proceedings of the SIGCHI Conference on Human Factors in Computing Systems*, (Boston, MA), 1151–1160. 10.1145/1518701.1518877

[B28] Duguid M. M. Goncalo J. A. (2012). Living large: the powerful overestimate their own height. *Psychol. Sci.* 23 36–40. 10.1177/0956797611422915 22173738

[B29] Dunn D. S. Burcaw S. (2013). Disability identity: exploring narrative accounts of disability. *Rehabil. Psychol.* 58 148–157. 10.1037/a0031691 23437994

[B30] Dunn R. A. Guadagno R. E. (2012). My avatar and me: gender and personality predictors of avatar-self discrepancy. *Comp. Human Behav.* 28 97–106. 10.1016/j.chb.2011.08.015

[B31] Epstein S. (2013). “The nature of anxiety with emphasis upon its relationship to expectancy,” in *Anxiety: Current Trends in Theory and Research*, ed. Spielberger C. D. (Amsterdam: Elsevier), 292–337.

[B32] Ferrer-Garcia M. Porras-Garcia B. González-Ibañez C. Gracia-Blanes M. Vilalta-Abella F. Pla-Sanjuanelo J. (2017). Does owning a “fatter” virtual body increase body anxiety in college students? *Annu. Rev. Cyber Ther. Telemed.* 15 147–153.

[B33] Fox J. Bailenson J. Binney J. (2009). Virtual experiences, physical behaviors: the effect of presence on imitation of an eating avatar. *Presence Teleoper. Virtual Environ.* 18 294–303. 10.1162/pres.18.4.294 32495221

[B34] Franke N. Schreier M. Kaiser U. (2010). The “I designed it myself” effect in mass customization. *Manag. Sci.* 56 125–140. 10.1287/mnsc.1090.1077 19642375

[B35] Gonnerman M. E. Jr. Parker C. P. Lavine H. Huff J. (2000). The relationship between self-discrepancies and affective states: the moderating roles of self-monitoring and standpoints on the self. *Pers. Soc. Psychol. Bull.* 26 810–819. 10.1177/0146167200269006

[B36] Grubb E. L. Grathwohl H. L. (1967). Consumer self-concept, symbolism and market behavior: a theoretical approach. *J. Mar.* 31 22–27. 10.2307/1249461

[B37] Haverinen A. (2016). “In-game and out-of-game mourning: on the complexity of grief in virtual worlds,” in *Mediating and Remedeating Death*, eds Christensen D. R. Sandvik K. (New York, NY: Ashgate), 154–178.

[B38] Hayes A. F. (2012). PROCESS: A Versatile Computational Tool for Observed Variable Mediation, Moderation, and Conditional Process Modeling (White paper). Available online at: http://www.afhayes.com/public/process2012.pdf (accessed June 13, 2019).

[B39] Hayes A. F. (2015). An index and test of linear moderated mediation. *Multivariate Behav. Res.* 50 1–22. 10.1080/00273171.2014.962683 26609740

[B40] Hefner D. Klimmt C. Vorderer P. (2007). “Identification with the player character as determinant of video game enjoyment,”,” in *International Conference on Entertainment Computing 2007*, eds Ma L. Nakatsu R. Rauterberg M. (Berlin: Springer), 39–48. 10.1007/978-3-540-74873-1_6

[B41] Higgins E. T. (1987). Self-discrepancy: a theory relating self and affect. *Psychol. Rev.* 94:319 10.1037/0033-295X.94.3.319 3615707

[B42] Higgins E. T. (1989). Self-discrepancy theory: what patterns of self-beliefs cause people to suffer? *Adv. Exp. Soc. Psychol.* 22 93–136. 10.1016/S0065-2601(08)60306-8

[B43] Higgins E. T. Klein R. Strauman T. (1985). Self-concept discrepancy theory: a psychological model for distinguishing among different aspects of depression and anxiety. *Soc. Cog.* 3 51–76. 10.1521/soco.1985.3.1.51

[B44] Hitchens C. (2014). *Mortality.* New York, NY: Twelve.

[B45] Hoffner C. Buchanan M. (2005). Young adults’ wishful identification with television characters: the role of perceived similarity and character attributes. *Media Psychol.* 7 325–351. 10.1207/S1532785XMEP0704_2

[B46] Hoffner C. A. Cohen E. L. (2012). Responses to obsessive compulsive disorder on Monk among series fans: parasocial relations, presumed media influence, and behavioral outcomes. *J. Broadcast. Elec. Media* 56 650–668. 10.1080/08838151.2012.732136

[B47] Hudson I. Hurter J. (2016). “Avatar types matter: review of avatar literature for performance purposes,” in *Proceedings of the International Conference on Virtual, Augmented and Mixed Reality*, Vol. 9740(Toronto), 14–21. 10.1007/978-3-319-39907-2_2

[B48] Jain S. L. Lynch J. E. (2011). Survival odds: mortality in corporate time. *Curr. Anthropol.* 52 545–555. 10.1086/656795

[B49] Jin S. A. A. (2012). Self-discrepancy and regulatory fit in avatar-based exergames. *Psychol. Rep.* 111 697–710. 10.2466/06.07.21.PR0.111.6.697-710 23402039

[B50] Kaiser U. Schreier M. Janiszewski C. (2017). The self-expressive customization of a product can improve performance. *J. Mar. Res.* 54 816–831. 10.1509/jmr.14.0293 11670861

[B51] Kalisch R. Wiech K. Critchley H. D. Seymour B. O’Doherty J. P. Oakley D. A. (2005). Anxiety reduction through detachment: subjective, physiological, and neural effects. *J. Cogn. Neurosci.* 17 874–883. 10.1162/0898929054021184 15969906

[B52] Kalyanaraman S. Sundar S. S. (2006). The psychological appeal of personalized content in web portals: does customization affect attitudes and behavior? *J. Comm.* 56 110–132. 10.1111/j.1460-2466.2006.00006.x

[B53] Kalyanaraman S. Wojdynski B. W. (2015). “Affording control: how customization, interactivity, and navigability affect psychological responses to technology,” in *The Handbook of the Psychology of Communication Technology*, ed. Shyam Sundar S. (Hoboken, NJ: John Wiley and Sons, Ltd), 425–444. 10.1002/9781118426456.ch19

[B54] Kim C. Lee S. G. Kang M. (2012). I became an attractive person in the virtual world: users’ identification with virtual communities and avatars. *Comp. Human Behav.* 28 1663–1669. 10.1016/j.chb.2012.04.004

[B55] Kim K. Schmierbach M. G. Chung M. Y. Fraustino J. D. Dardis F. Ahern L. (2015). Is it a sense of autonomy, control, or attachment? exploring the effects of in-game customization on game enjoyment. *Comp. Human Behav.* 48 695–705. 10.1016/j.chb.2015.02.011

[B56] Kim Y. Sundar S. S. (2012). Visualizing ideal self vs. actual self through avatars: impact on preventive health outcomes. *Comp. Human Behav.* 28 1356–1364. 10.1016/j.chb.2012.02.021

[B57] Kolko B. E. (1999). Representing bodies in virtual space: the rhetoric of avatar design. *Info. Soc.* 15 177–186. 10.1080/019722499128484

[B58] Konijn E. A. Hoorn J. F. (2005). Some like it bad: testing a model for perceiving and experiencing fictional characters. *Media Psychol.* 7 107–144. 10.1207/S1532785XMEP0702_1

[B59] Kromand D. (2007). “Avatar categorization,” in *Proceedings of the 2007 DiGRA International Conference: Situated Play*, (Tokyo: The University of Tokyo).

[B60] Large B. MacLeod C. Clarke P. J. Notebaert L. (2016). It’s all about control: memory bias in anxiety is restricted to threat cues that signal controllable danger. *J. Exp. Psychopathol.* 7 190–204. 10.5127/jep.048515

[B61] Larson P. C. Boyle E. S. Boaz M. E. (1984). Relationship of self-concept to age, disability, and institutional residency. *The Gerontologist* 24, 401–407. 10.1093/geront/24.4.401 6479654

[B62] Lazarus R. S. (1991). *Emotion and Adaptation.* Oxford: Oxford University Press.

[B63] Legare C. H. Lane J. D. Evans E. M. (2013). Anthropomorphizing science: how does it affect the development of evolutionary concepts? *Merrill Palmer Q.* 59 168–197. 10.1353/mpq.2013.0009

[B64] Li D. D. Liau A. K. Khoo A. (2013). Player–Avatar Identification in video gaming: concept and measurement. *Comp. Human Behav.* 29 257–263. 10.1016/j.chb.2012.09.002

[B65] Lilienfeld S. O. Turner S. M. Jacob R. G. (1993). Anxiety sensitivity: an examination of theoretical and methodological issues. *Adv. Behav. Res. Ther.* 15 147–183. 10.1016/0146-6402(93)90019-X

[B66] Liu Y. (2003). Developing a scale to measure the interactivity of websites. *J. Advert. Res.* 43 207–216. 10.1017/S0021849903030204

[B67] Lo S. K. Wang C. C. Fang W. (2005). Physical interpersonal relationships and social anxiety among online game players. *Cyberpsychol. Behav.* 8 15–20. 10.1089/cpb.2005.8.15 15738689

[B68] MacLeod C. Mathews A. (1988). Anxiety and the allocation of attention to threat. *Q. J. Exp. Psychol.* 40 653–670. 10.1080/14640748808402292 3212208

[B69] Mancini T. Sibilla F. (2017). Offline personality and avatar customisation. Discrepancy profiles and avatar identification in a sample of MMORPG players. *Comput. Human Behav.* 69 275–283. 10.1016/j.chb.2016.12.031

[B70] Mancini T. Imperato C. Sibilla F. (2019). Does avatar’s character and emotional bond expose to gaming addiction? Two studies on virtual self-discrepancy, avatar identification and gaming addiction in massively multiplayer online role-playing game players. *Comput. Human Behav.* 92 297–305. 10.1016/j.chb.2018.11.007

[B71] Mann M. M. Hosman C. M. Schaalma H. P. De Vries N. K. (2004). Self-esteem in a broad-spectrum approach for mental health promotion. *Health Edu. Res.* 19 357–372. 10.1093/her/cyg041 15199011

[B72] Marathe S. Sundar S. S. (2011). “What drives customization?: control or identity?,” in *Proceedings of the SIGCHI Conference on Human Factors in Computing Systems*, (ACM), (Vancouver, BC), 781–779.

[B73] Markus H. (1977). Self-schemata and processing information about the self. *J. Pers. Soc. Psychol.* 35:63 10.1037/0022-3514.35.2.63

[B74] Marshall N. (2008). “Borders and bodies in city of heroes:(Re) imaging american identity post 9/11,” in *Computer Games as a Sociocultural Phenomenon*, eds Jahn-Sudmann A. Stockmann R. (London: Palgrave Macmillan)), 140–149. 10.1057/9780230583306_14

[B75] Marteau T. M. Bekker H. (1992). The development of a six−item short−form of the state scale of the Spielberger State—Trait Anxiety Inventory (STAI). *Br. J. Clin. Psychol.* 31 301–306. 10.1111/j.2044-8260.1992.tb00997.x 1393159

[B76] McNally R. J. (1989). Is anxiety sensitivity distinguishable from trait anxiety? Reply to Lilienfeld, Jacob, and Turner (1989). *J. Abnormal Psychol.* 98 193–194. 10.1037/0021-843X.98.2.193 2708664

[B77] Mehroof M. Griffiths M. D. (2010). Online gaming addiction: the role of sensation seeking, self-control, neuroticism, aggression, state anxiety, and trait anxiety. *Cyberpsychol. Behav. Soc. Netw.* 13 313–316. 10.1089/cyber.2009.0229 20557251

[B78] Moser D. K. (2007). “The rust of life”: impact of anxiety on cardiac patients. *Am. J. Critical Care* 16 361–369. 10.4037/ajcc2007.16.4.361 17595368 PMC2668571

[B79] Mukherjee S. (2010). *The Emperor of All Maladies: A Biography of Cancer.* New York, NY: Simon and Schuster.

[B80] Norton M. I. Mochon D. Ariely D. (2012). The ‘IKEA effect’: when labor leads to love. *J. Consumer Psychol.* 22 453–460. 10.1016/j.jcps.2011.08.002

[B81] Nöth W. Bishara N. Neitzel B. (2008). *Mediale Selbstreferenz: Grundlagen und Fallstudien zu Werbung, Computerspiel und den Comics.* Cologne: Halem.

[B82] Nowak K. L. Fox J. (2018). Avatars and computer-mediated communication: a review of the definitions, uses, and effects of digital representations. *Rev. Comm. Res.* 6 30–53. 10.12840/issn.2255-4165.2018.06.01.01

[B83] Olstead R. (2002). Contesting the text: canadian media depictions of the conflation of mental illness and criminality. *Sociol. Health Illness* 24 621–643. 10.1111/1467-9566.00311

[B84] Peachey A. (2010). “The third place in Second Life: real life community in a virtual world,” in *Researching Learning in Virtual Worlds*, eds Peachey A. Livingstone D. Gillen J. Smith-Robbins S. (London: Springer), 91–110. 10.1007/978-1-84996-047-2_6

[B85] Price J. Sloman L. Gardner R. Gilbert P. Rohde P. (1994). The social competition hypothesis of depression. *Br. J. Psychiatry* 164 309–315. 10.1192/bjp.164.3.309 8199784

[B86] Przybylski A. K. Weinstein N. Murayama K. Lynch M. F. Ryan R. M. (2012). The ideal self at play: the appeal of video games that let you be all you can be. *Psychol. Sci.* 23 69–76. 10.1177/0956797611418676 22173739

[B87] Reddit (2017). *A Physical Manifestation of Borderline Personality Disorder (Artwork by Toby Allen). r/BPD.* Available online at: https://www.reddit.com/r/BPD/comments/6mac1k/a_physical_manifestation_of_borderline/ (accessed February 19, 2019).

[B88] Reeves B. Nass C. (1996). *The Media Equation: How People Respond to Computers, Television, and New Media Like Real People and Places.* Cambridge: MA: University Press.

[B89] Richards A. French C. C. Johnson W. Naparstek J. Williams J. (1992). Effects of mood manipulation and anxiety on performance of an emotional Stroop task. *Br. J. Psychol.* 83 479–491. 10.1111/j.2044-8295.1992.tb02454.x 1486362

[B90] Rotter J. B. (1966). Generalized expectancies for internal versus external control of reinforcement. *Psychol. Mono.* 80 1–28. 10.1037/h0092976 5340840

[B91] Rule W. R. Traver M. D. (1983). Test-retest reliabilities of State-Trait Anxiety Inventory in a stressful social analogue situation. *J. Pers. Assess.* 47 276–277. 10.1207/s15327752jpa4703_8 6886960

[B92] Schafer R. B. Wickrama K. A. S. Keith P. M. (1996). Self-concept disconfirmation, psychological distress, and marital happiness. *J. Marriage Fam.* 58 167–177. 10.2307/353385

[B93] Schlenker B. R. (1985). *The Self and Social Life.* New York, NY: McGraw-Hill College.

[B94] Schmukle S. C. Egloff B. (2006). Assessing anxiety with extrinsic Simon tasks. *Exp. Psychol.* 53 149–160. 10.1027/1618-3169.53.2.149 16909940

[B95] Schnurr B. Scholl-Grissemann U. (2015). Beauty or function? How different mass customization toolkits affect customers’ process enjoyment. *J. Consum. Behav.* 14 335–343. 10.1002/cb.1524

[B96] Schultze U. (2010). Embodiment and presence in virtual worlds: a review. *J. Info. Tech.* 25 434–449. 10.1057/jit.2010.25

[B97] Slater M. (2009). Inducing illusory ownership of a virtual body. *Front. Neurosci* 3 214–220. 10.3389/neuro.01.029.2009 20011144 PMC2751618

[B98] Spanlang B. Normand J.-M. Borland D. Kilteni K. Giannopoulos E. Pomés A. (2014). How to build an embodiment lab: achieving body representation illusions in virtual reality. *Front. Robot. AI* 1, 1–22. 10.3389/frobt.2014.00009

[B99] Spielberger C. D. Gorsuch R. L. Lushene R. E. (1970). *Manual for the State-Trait Anxiety Inventory.* Palo Alto, CA: Consulting psychologists press.

[B100] Stewart S. Hansen T. S. Carey T. A. (2010). Opportunities for people with disabilities in the virtual world of Second Life. *Rehabil. Nurs.* 35 254–259. 10.1002/j.2048-7940.2010.tb00056.x 21140720

[B101] Strauman T. J. (1996). Stability within the self: a longitudinal study of the structural implications of self-discrepancy theory. *J. Pers. Soc. Psychol.* 71:1142. 10.1037/0022-3514.71.6.1142 8979383

[B102] Sundar S. S. (2008). “The MAIN model: a heuristic approach to understanding technology effects on credibility,” in *Digital Media, Youth, and Credibility*, eds Flanagin A. J. Metzger M. J. (Cambridge, MA: The MIT Press), 73–100. 10.1162/dmal.9780262562324.073

[B103] Taylor T. L. (2002). “Living digitally: embodiment in virtual worlds,” in *The social life of avatars*, ed. Schroeder R. (London: Springer), 40–62. 10.1007/978-1-4471-0277-9_3

[B104] Thoits P. A. (2013). “Self, identity, stress, and mental health,” in *Handbook of the Sociology of Mental Health*, eds Aneshensel C. S. Phelan J. C. (Dordrecht: Springer Netherlands), 357–377. 10.1007/978-94-007-4276-5_18

[B105] Tregaskis C. (2002). Social model theory: the story so far. *Disabil. Soc.* 17 457–470. 10.1080/09687590220140377

[B106] Turkay S. Adinolf S. (2010). Free to be me: a survey study on customization with World of Warcraft and City of Heroes/Villains players. *Procedia Soc. Behav. Sci.* 2 1840–1845. 10.1016/j.sbspro.2010.03.995

[B107] Van Der Heide B. Schumaker E. M. Peterson A. M. Jones E. B. (2013). The Proteus effect in dyadic communication: examining the effect of avatar appearance in computer-mediated dyadic interaction. *Comm. Res.* 40 838–860. 10.1177/0093650212438097

[B108] Van Looy J. Courtois C. De Vocht M. De Marez L. (2012). Player identification in online games: validation of a scale for measuring identification in MMOGs. *Media Psychol.* 15 197–221. 10.1080/15213269.2012.674917

[B109] Vasalou A. Joinson A. N. (2009). Me, myself and I: the role of interactional context on self-presentation through avatars. *Comput. Human Behav.* 25 510–520. 10.1016/j.chb.2008.11.007

[B110] Vorderer P. Knobloch S. (2000). *Media Entertainment: The Psychology of Its Appeal.* New York, NY: Routledge.

[B111] Waddell T. F. Sundar S. S. Auriemma J. (2015). Can customizing an avatar motivate exercise intentions and health behaviors among those with low health ideals? *Cyberpsychol. Behav. Soc. Netw.* 18 687–690. 10.1089/cyber.2014.0356 26406804

[B112] Wenz K. (2014). “Death,” in *The Routledge Companion to Video Game Studies*, eds Wolf M. J. P. Perron B. (New York, NY: Routledge).

[B113] Williams J. M. G. Watts F. N. MacLeod C. Mathews A. (1988). *Cognitive Psychology and Emotional Disorders.* Hoboken, NJ: John Wiley and Sons.

[B114] World Health Organization [WHO] (2001). *The World Health Report.* Available online at: http://www.who.int/whr/2001/en/ (accessed January 12, 2019).

[B115] Wu Y. Babu S. V. Armstrong R. Bertrand J. W. Luo J. Roy T. (2014). Effects of virtual human animation on emotion contagion in simulated inter-personal experiences. *IEEE Trans. Vis. Comput. Graph.* 20 626–635. 10.1109/TVCG.2014.19 24650990

[B116] Yee N. Bailenson J. (2007). The Proteus effect: the effect of transformed self−representation on behavior. *Human Comm. Res.* 33 271–290. 10.1111/j.1468-2958.2007.00299.x

